# Cuproptosis-associated lncRNAs discern prognosis and immune microenvironment in sarcoma victims

**DOI:** 10.3389/fcell.2022.989882

**Published:** 2022-12-16

**Authors:** Binxiang Chu, Haihong Zheng, Xiaohe Zheng, Xingbing Feng, Zhenghua Hong

**Affiliations:** ^1^ Department of Orthopedic, Taizhou Hospital of Zhejiang Province Affiliated to Wenzhou Medical University, Linhai, China; ^2^ Department of Pathology, Taizhou Hospital of Zhejiang Province Affiliated to Wenzhou Medical University, Linhai, China

**Keywords:** cuproptosis, lncRNA, sarcoma, tumor immune microenvironment, immunotherapy, prognosis

## Abstract

Cuproptosis is a fresh form of the copper-elesclomol-triggered, mitochondrial tricarboxylic acid (TCA) dependent cell death. Yet, the subsumed mechanism of cuproptosis-associated lncRNAs in carcinoma is not wholly clarified. Here, We appraised 580 cuproptosis-associated lncRNAs in sarcoma and thereafter construed a module composing of 6 cuproptosis lncRNAs, entitled CuLncScore, utilizing a machine learning methodology. It could outstandingly discern the prognosis of patients in parallel with discriminating tumor immune microenvironment traits. Moreover, we simulate the classification system of cuproptosis lncRNAs by unsupervised learning method to facilitate differentiation of clinical denouement and immunotherapy modality options. Notably, Our Taizhou cohort validated the stability of CuLncScore and the classification system. Taking a step further, we checked these 6 cuproptosis lncRNAs by Quantitative real-time polymerase chain reaction (qRT-PCR) to ascertain their authenticity. All told, our investigations highlight that cuproptosis lncRNAs are involved in various components of sarcoma and assist in the formation of the tumor immune microenvironment. These results provide partial insights to further comprehend the molecular mechanisms of cuproptosis lncRNAs in sarcoma and could be helpful for the development of personalized therapeutic strategies targeting cuproptosis or cuproptosis lncRNAs.

## Introduction

Sarcoma is not a singular entity, but a set of well over 100 subtypes of malignant growths derived from the wider mesenchymal stroma of bone and soft tissue, with a spectrum of mutable biological and clinical traits (Gamboa et al., 2020). Given the prevalence, they represented 20% of solid malignancies in pediatrics but were only responsible for less than 1% of cancers in adults (Baldi et al., 2022). Surgery and additional chemo or radiotherapy are the ongoing standards of care (Singh et al., 2020; Damerell et al., 2021). Due to their heterogeneous nature and perceptiveness to contemporary treatments, their clinical management is extremely challenging. Overall, the ultimate prognosis of sarcomas patients is not favorable (Pestana et al., 2022). Therefore exploring new biomarkers and their intrinsic specific biological properties to identify poor prognosis and provide therapeutic targets is a tangible approach.

Cuproptosis, a novel form of the copper-elesclomol-triggered, mitochondrial tricarboxylic acid (TCA) dependent cell death, occurs by a mechanism independent of well-known apoptosis and pyroptosis (Tsvetkov et al., 2022). Juxtaposing copper impacts cell fate and is engaged in carcinogenesis (Ge et al., 2022), cuproptosis is bound to trigger a fresh wave of investigation in oncology. It was noted in the latest articles that FDX1 (Tsvetkov et al., 2019), a key gene for cuproptosis, is firmly associated with the metabolism of the three major nutrients (glucose, fatty acids, and amino acids) in the lung adenocarcinoma and its down-regulated expression could be an indicator of poor prognosis (Zhang et al., 2021).

lncRNAs are non-protein-coding RNA species with multifarious bio functions that have been documented to be dysregulated in a wide range of pathologies and to be pivotal agents in the ontogenesis of carcinogenesis (Heward and Lindsay, 2014; Ghafouri-Fard et al., 2021; Goodall and Wickramasinghe, 2021). In sarcoma, certain lncRNAs are inextricably entwined with malignant cell proliferation and migration, making them well-suited as relevant biosignatures and therapeutic targets (Marques Howarth et al., 2014; Lin et al., 2021; Aryee et al., 2022). Moreover, several outstanding lncRNA-based models have been constructed to forecast the prognosis of certain tumor cases, which dramatically enriches the potential application of lncRNA for cancer (Guo et al., 2022; Liu et al., 2022; Xing et al., 2022). Here is the question, what is the possible ramification of cuproptosis-associated lncRNAs in sarcoma and could it be a potential biomarker. Here is the question, what is the possible ramification of cuproptosis-associated lncRNAs in sarcoma and could it be a potential biomarker.

To systematically understand cuproptosis lncRNAs and unravel their mystery in sarcoma, we unearthed genomic information of TCGA-SARC samples in an attempt to disentangle the presumable role of cuproptosis lncRNAs in sarcoma. We were excited to detect a significant impact of cuproptosis lncRNAs on the tumor microenvironment. Moreover, We constructed a new risk-prognosis model, called CuLncScore, to facilitate the easy assessment of patient prognosis and therapeutic response. Moving further, we partitioned patients into 4 clusters to delineate their variable immune status and prognosis. Of notable is that the CuLncScore and categories were validated in our Taizhou cohort. Our study provides significant insight into the function of cuproptosis lncRNAs in sarcoma.

## Materials and methods

### Authentication of cuproptosis-associated lncRNA

Peter et al. proposed new insights into cuproptosis, from which we extracted 12 key genes, covering FDX1, LIAS, LIPT1, DLD, DLAT, PDHA1, PDHB, MTF1, GLS, CDKN2A, ATP7B, and SLC31A1, named cuproptosis genes (Tsvetkov et al., 2022). Pearson correlation was applied to compute the correlation between lncRNAs and cuproptosis genes, where lncRNAs with absolute |correlation coefficients| > 0.4 and *p* < .001 were accepted as cuproptosis-associated lncRNAs.

### CuLncScore model construction and cuproptosis lncRNA clusters identification

In this investigation, we enrolled RNA sequencing data, SNV data, CNV data, and clinical data from the TCGA-SARC cohort, Data above were divided equally into train and test groups based on random assignment. To be first, a cuproptosis lncRNAs score model, entitled CuLncScore, was constructed using LASSO regression. AC131056.3, AP001476.2, CTC-453G23.8, CTC-550B14.7, CTD-2651B20.7, and RP11-351I21.11 opted for model configuration. Based on the linear combination of gene expression values and regression coefficients, risk scores were calculated for SARC patients. The formula was as follows:

$$Risk\;score=\overset{n}{\underset{i=1}{\sum }}Coef_{i}\times \mathrm{Exp}r_{i}$$

Exp = gene expression value. Coe = regression coefficient. Subsequently, univariate and multivariate Cox regression analyses were performed on the CuproScore model. The “rms” R package was on hand to create a nomogram. Meanwhile, unsupervised clustering analysis was used to identify cuproptosis lncRNAs phenotypes in SARC to categorize patients. “ConsensusClusterPlus” R package was designed to run each step. In particular, the “Maftools” R package and GISTIC 2.0 online software were implemented for the genetic characterization of the genome. Separately, the osteosarcoma cohort from the TARGET database served as an external validation.

### Enrichment and immune infiltration analysis

The gene set enrichment analysis (GSEA) was performed to check potential biological processes in high and low-risk groups. The “CIBERSORT” R package was used to estimate the proportion of immune cells.

### Transcriptomic sequencing of Taizhou cohort

We retrospectively enrolled 40 patients who had surgical treatment at Taizhou Hospital from 1 February 2014, to 30 December 2021, which we named the Taizhou cohort. The study followed the guiding principles of the Declaration of Helsinki and was approved by the ethical review committee of Taizhou Hospital. All participants gave written informed consent. A total of 12 tumor samples (consisting of 3 undifferentiated sarcomas, 3 rhabdomyosarcomas, 3 leiomyosarcomas, and 3 endometrial stromal sarcomas) and 6 paracancerous normal control samples were submitted for transcriptome sequencing. Total RNA was purified from FFPE samples and only samples with DV200 ≥ 30% were selected for subsequent RNA sequence analysis. See our previous report for more details on the method (Shi et al., 2021).

### Quantitative real-time polymerase chain reaction (qRT-PCR)

After paraffin tissue was dewaxed, RNA was isolated by Trizol reagent (Invitrogen, United States). Then, complementary DNA (cDNA) was synthesized *via* PrimeScriptTM RT kit (Takara). qRT-PCR analysis was run on the SYBR Premix Ex Taq (Takara). Normalization of all expression databases to GAPDH (as an endo-controller gene) using the 2^−ΔΔCT^ method. Primer sequences referred to the following.

AC131056.3_Forward: 5′- GCT​GTC​AAA​TGA​GGC​AGG​TTG-3'; Reverse: 5′-GTA​AGA​GCC​TGA​TGC​GTG​GA-3'. AP001476.2_Forward: 5′-GTT​TCA​TAA​GTG​AAA​TGC​CTG​CT-3'; Reverse: 5′-ACC​CTC​ACT​GGA​TAC​ACC​AAA​A-3'. CTC-453G23.8_Forward: 5′-ATC​TTT​GGC​GTG​TCA​GCA​CT-3'; Reverse: 5′-CCA​AGT​CTC​CAG​ACT​CTT​GCT​T -3'. CTC-550B14.7_Forward: 5′-TCT​TCT​GGT​TGT​TCT​GAA​GGG-3'; Reverse: 5′-ACT​CTT​CCT​TTG​GGG​AAG​CAA-3'. CTD-2651B20.7_Forward: 5′-TGA​TGA​GGG​AGT​CGC​AAG​CA-3'; Reverse: 5′-ACA​AGT​CAG​GCT​TCT​TGA​CCC-3'. RP11-351I21.11_Forward: 5′-CAT​TCT​GGT​GGC​CGA​GAG​AC-3'; Reverse: 5′-TTG​GGC​TGA​GAT​GGC​TAG​TAT​T-3'. GAPDH_Forward: 5′- GGA​GCG​AGA​TCC​CTC​CAA​AAT-3'; Reverse: 5′- GGC​TGT​TGT​CAT​ACT​TCT​CAT​GG-3'.

### Statistical analysis

R software (version 3.6.1) was used for all statistical analyses. Wilcoxon test was used for comparison between groups if not otherwise stated. Kruskal test was employed for overall comparison of multiple sets and Pearson correlation coefficient was for correlation analysis.

## Results

### Authentication and preliminary insight of cuproptosis LncRNAs

**FIGURE 1:**
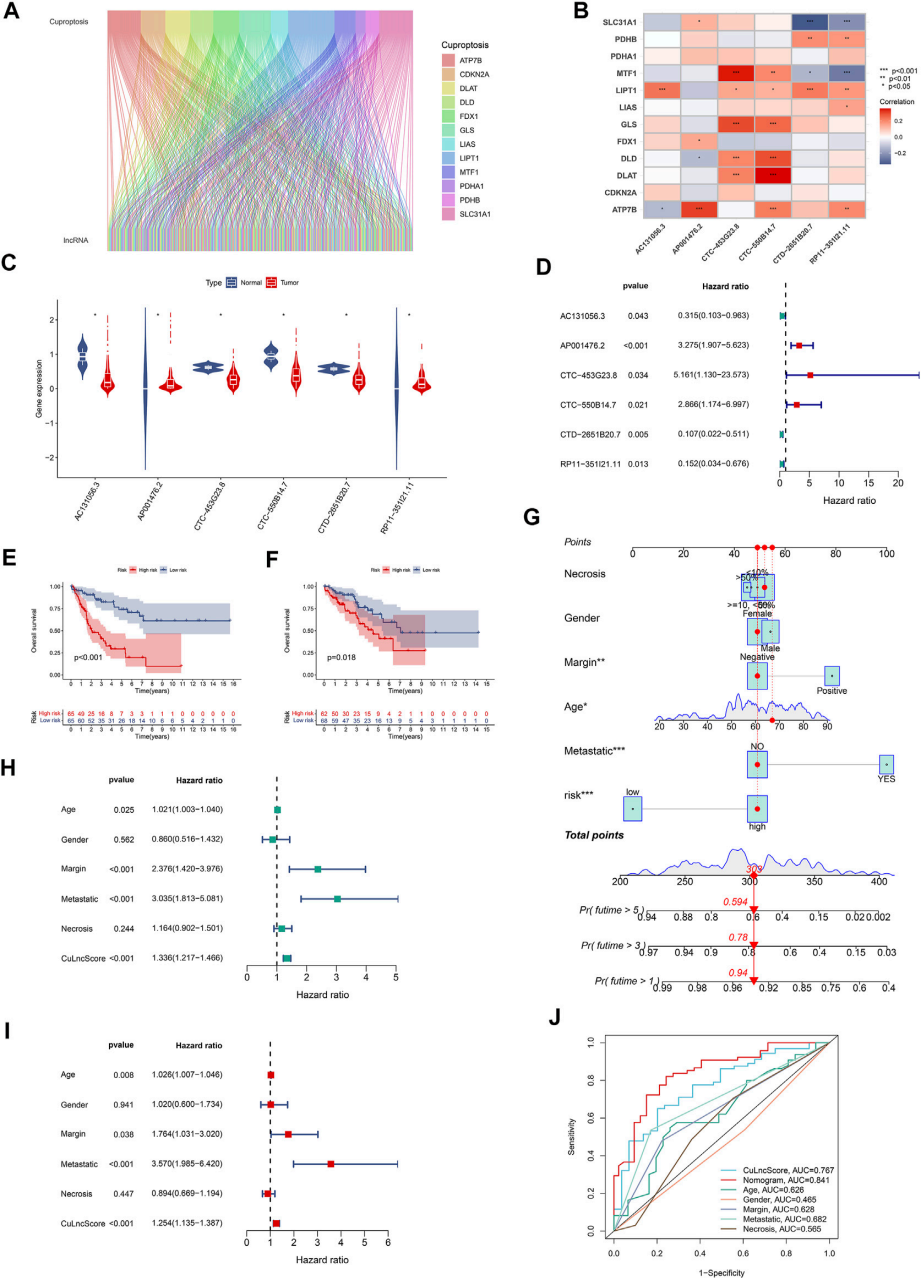
Clinical relevance of CuLncScore. **(A)** The lncRNAs that are co-expressed with cuproptosis-related genes in the TCGA-SARC cohort are labeled cuproptosis lncRNAs. **(B)** The co-expression correlation of six cuproptosis lncRNAs for constructing the CuLncScore model with cuproptosis genes. **(C)** Variable expression of cuproptosis lncRNAs in TCGA-SARC. **(D)** Cox regression analysis of TCGA-SARC with cuproptosis lncRNAs. Kaplan-Meier Curves for differential survival in high and low-risk groups in the train **(E)** and test **(F)** cohort. **(G)** A nomogram for predicting the 1, 3, and 5 years survival rates of patients. Univariate **(H)** and multivariate **(I)** Cox regressions confirm CuLncScore as an independent prognostic factor. **(J)** ROC curves for different risk factors. *p < .05, **p < 0.01, ***p < .001.

Cuproptosis is a fresh conception, and the underlying role of cuproptosis-associated lncRNAs in sarcoma captured our attention particularly well, both in terms of their expression patterns and potential prognostic impact. For starters, in TCGA-SARC, we authenticated 580 cuproptosis lncRNAs by co-expression analysis algorithm (Figure 1A). As a further step, a risk scoring tool with 6 cuproptosis lncRNAs (including AC131056.3, AP001476.2, CTC-453G23.8, CTC-550B14.7, CTD-2651B20.7, and RP11-351I21.11), dubbed CuLncScore, was configured by machine learning (lasso regression analysis). For details, the risk score of the CuLncScore model = AC131056.3 × -1.209 + AP001476.2 × 1.402+CTC-550B14.7 × 1.057+CTD-2651B20.7 × -1.517 + RP11-351I21.11 × -1.121. These lncRNAs were variably linked to cuproptosis genes, with CTC-453G23.8 and CTC-550B14.7 positively corresponding to at least five cuproptosis genes (Figure 1B). In the initial divergent analysis, AC131056.3, CTC-453G23.8, CTC-550B14.7, and CTD-2651B20.7 were markedly hypo-regulated in tumor tissues, while AP001476.2 and RP11-351I21.11 were dramatically hyper-regulated (Figure 1C). Strikingly, COX regression analysis unveiled AC131056.3, CTD-2651B20.7, and RP11-351I21.11 as protective agents, while AP001476.2, CTC-453G23.8, CTC-550B14.7 as risk drivers (Figure 1D).

### Clinical significance of CuLncScore

**FIGURE 2:**
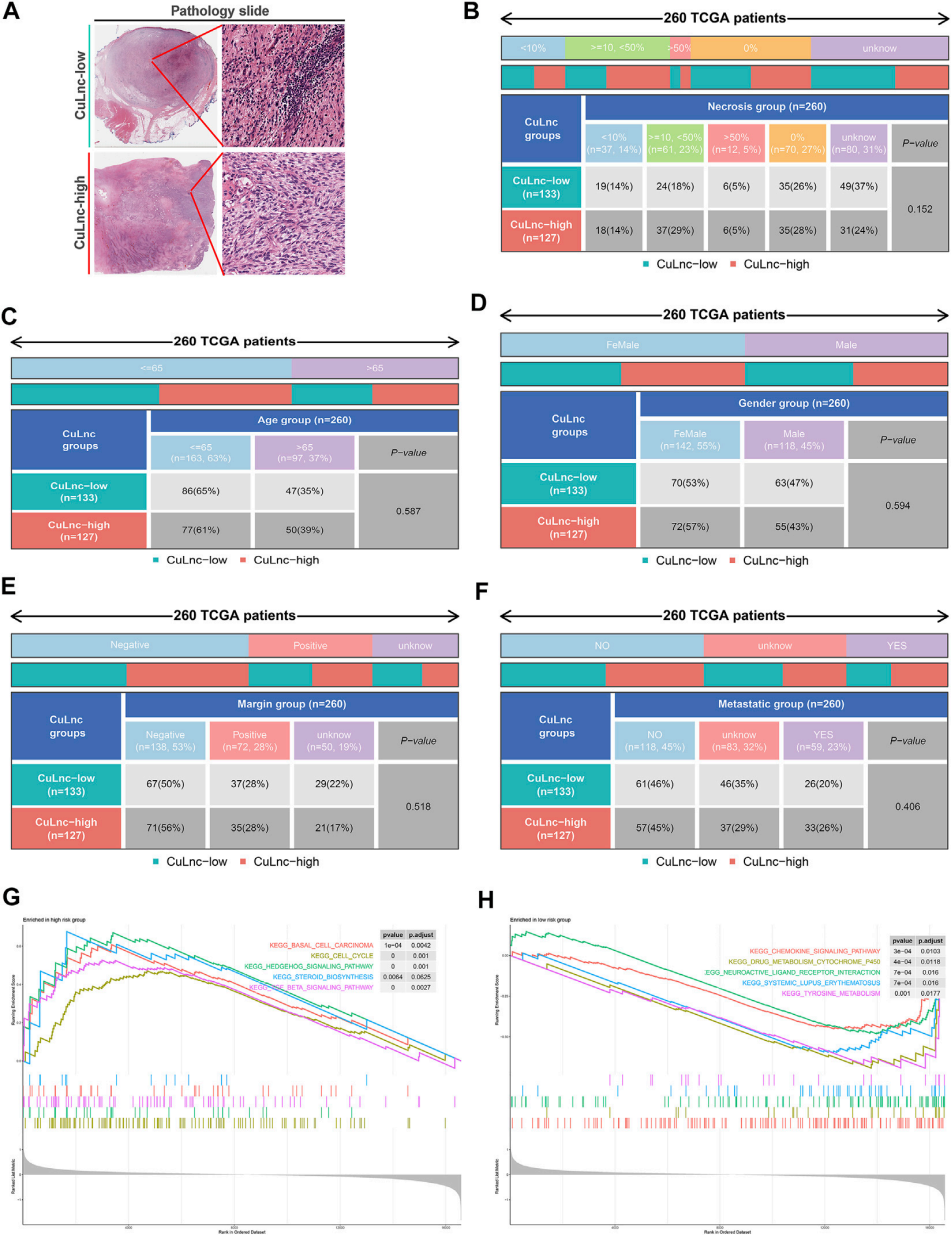
The clinicopathological and biological characteristics of the CuLncScore grouping. **(A)** Representative images of the pathological HE staining of the high or low-risk group from the TCGA dataset. Necrosis **(B)**, Age **(C)**, Gender **(D)**, Margin **(E)**, and Metastatic **(F)** characteristics of high and low-risk groups in TCGA-SARC. KEGG pathway enrichment analysis in the high-risk group **(G)** and low-risk group **(H)**.

To check the stability of the model, we randomly grouped the TCGA-SARC cohort into two equally, named the train group and the test group. Soon after, based on the median score, patients were ranked as high-risk and low-risk, respectively. By Kaplan-Meier analysis, we showed that the CuLncScore could accurately predict prognosis, those with higher risk had a worse prognosis. This was confirmed in both the train group (Figure 1E) and test group (Figure 1F). In particular, through univariate (Figure 1H) and multivariate (Figure 1I) Cox analysis, we verified that the model could independently predict the prognosis of patients with SARC (TCGA Pathology Slide, Figure 2A). Moreover, to facilitate clinician assessment, we also constructed nomo plots for presenting convenient quantitative methods applicable to predict patients’ 1-year, 3-year, and 5-year overall survival (Figure 1G) and confirmed the better prognostic, predictive validity of nomo plots by the area under curves (Figure 1J). Then, the chart summarizes the clinicopathological characteristics of the high- and low-risk sets, touching upon age (Figure 2C), gender (Figure 2D), necrosis (Figure 2B), margin (Figure 2E) and metastatic (Figure 2F).

### Characterization of biological trajectories and genetics in the CuLncScore

**FIGURE 3:**
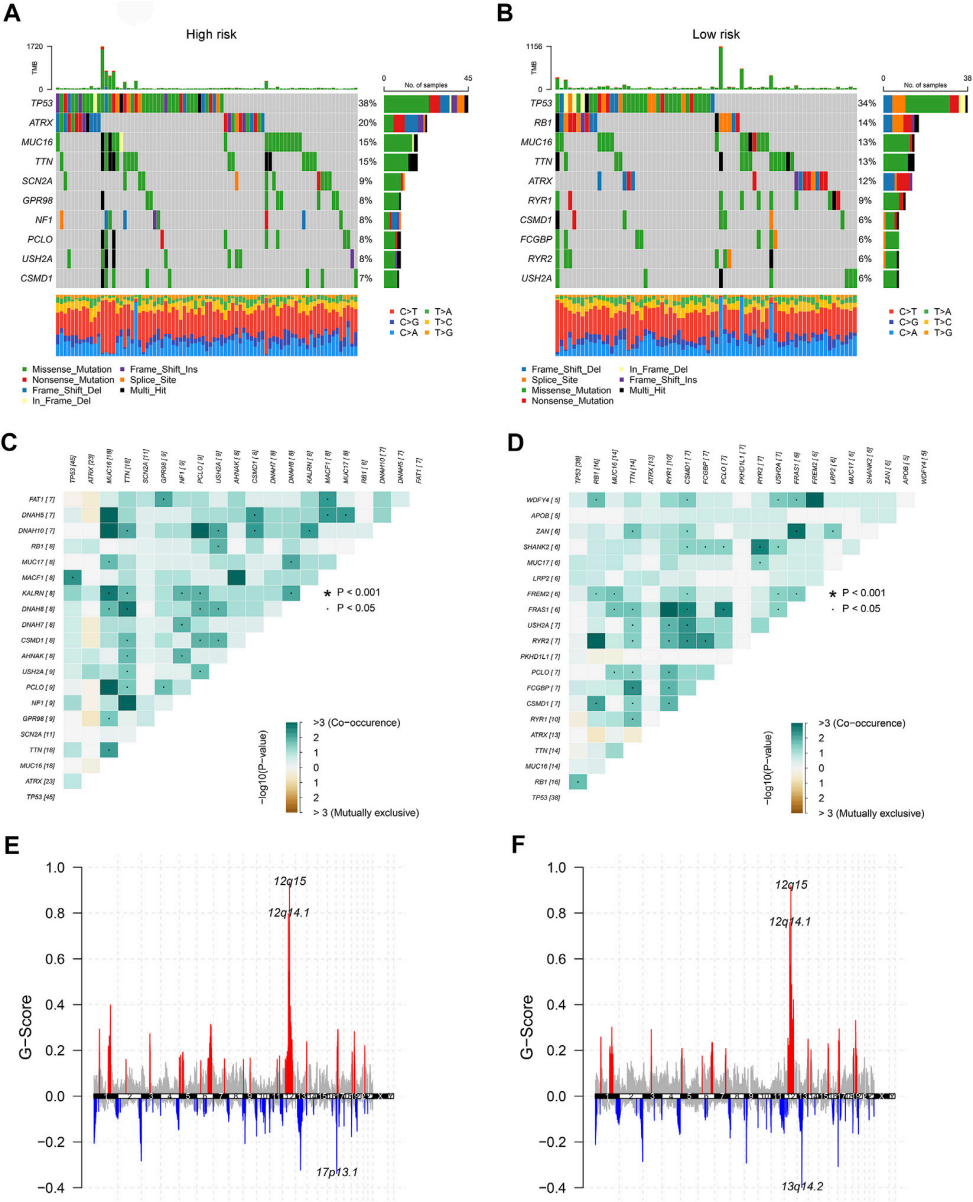
The genetic characteristics of the CuLncScore grouping. Somatic mutation characteristics of the high **(A)** and low **(B)** risk groups. The co-occurrence and mutually exclusive mutations of the differently mutated genes in the high **(C)** and low **(D)** risk groups. GISTIC 2.0 amplifications (red) and deletions (blue) in the high **(E)** and low **(F)** CuLncScore groups.

To dig deeper, we aimed to use GSEA to explore the differences in intrinsic molecular mechanisms comparing the high- and low-risk groups and revealed that pathways associated with the high-risk group included basal cell carcinoma, cell cycle, and hedgehog signaling pathway (Figure 2G), while pathways associated with the low-risk group included chemokine signaling pathway, drug metabolism cytochrome p450, and neuroactive ligand receptor interaction (Figure 2H). Next-generation sequencing (NGS) methods furnish multidimensional databanks driving the mutational universe of genes that may account for tumorigenesis mechanisms and heterogeneity. To further debunk the genetic variation in each of the two risk subgroups to understand their differentiated prognosis, we mutilated the SNV and CNV data which revealed the occurrence of different somatic mutations between the two. For case, the top ten mutated genes in the high-risk group were TP53, ATRX, MUC16, TTN, SCN2A, GPR98, NF1, PCLO, USH2A, and CSMD1 (Figure 3A), while those with low risk were TP53, RB1, MUC16, TTN, ATRX, RYR1, CSMD1, FCGBP, RYR2, and USH2A (Figure 3B). They share distinct somatic co-mutation profiles, with TP53 co-mutated with MACF1 in the high-risk group (Figure 3C) whereas TP53 co-mutated with RB1 in the low-risk one (Figure 3D). In addition, we informed that 17p13.1 deletion was the most prevalent genomic event in individuals at high risk (Figure 3E), whilst 13q14.2 deletion was reported in primarily the low-risk group (Figure 3F).

### Characterization of the immune microenvironment in the CuLncScore

**FIGURE 4:**
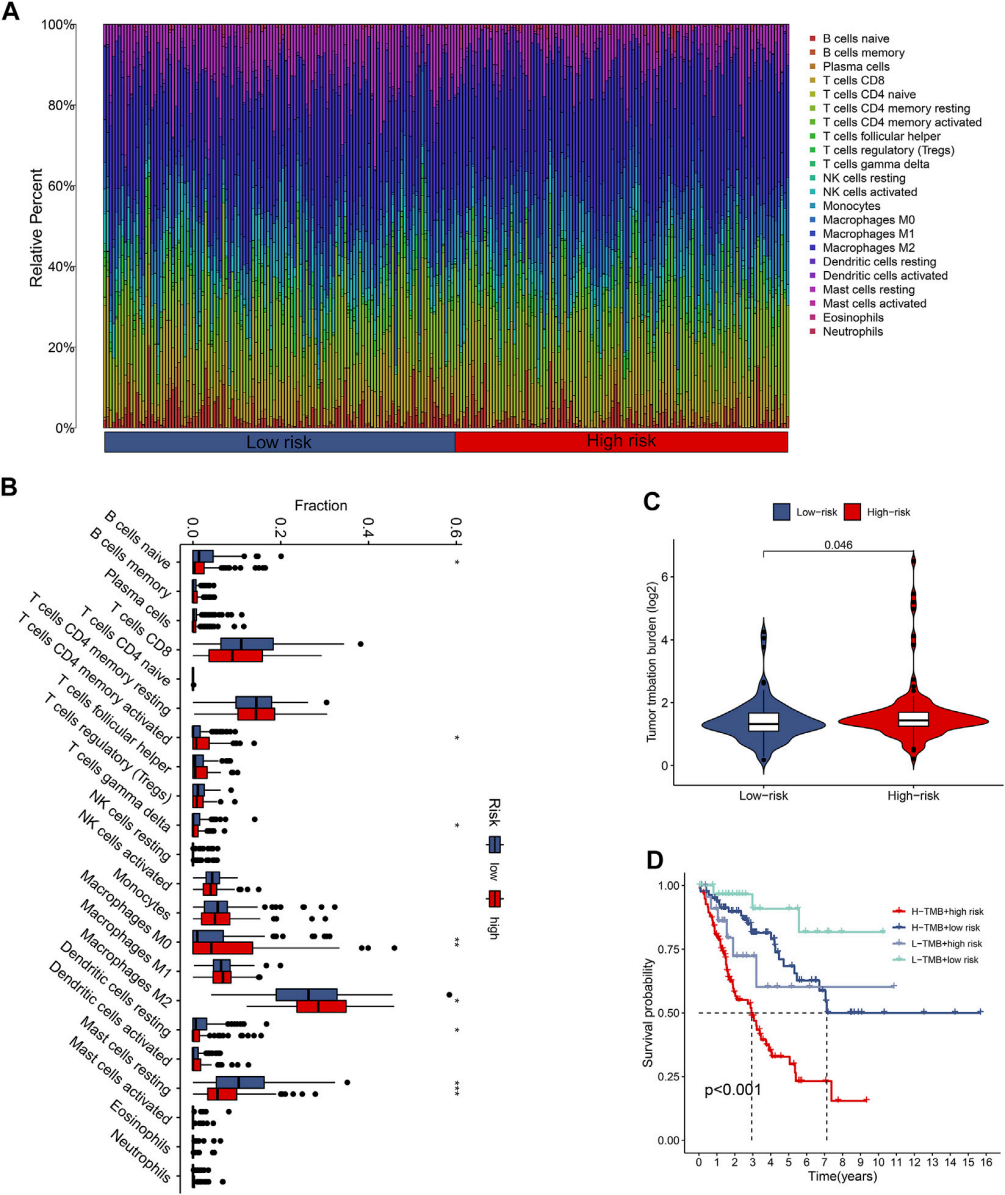
The immune landscape of the CuproScore grouping. **(A)** Histogram of immune cell content in high and low-risk groups. **(B)** Histogram of discrepancy immune infiltration of two groups. **(C)** Comparative tumor mutation burden (TMB) in the two groups. **(D)** Prognostic analysis of TMB in the different groups. *p < .05, **p < .01, ***p < .001.

The tumor immune microenvironment is firmly linked to patient prognosis and immunotherapeutic response (Baghban et al., 2020; Mattei et al., 2020). To further explore the potential relationship between the CuLncScore model and the immune system, we ran tests for the comparison of the ratios of immune cells in the two groups. Here, low-risk subjects had increased profusion of B cells naive, T cells gamma delta, and mast cells resting, compared with higher T cells CD4 memory activated infiltration in the high-risk cohort (Figure 4B). The histogram visually presented us with 22 immune cell ratios (Figure 4A). The present study establishes that TMB is closely tied to prognosis, with high TMB hinting at a worse prognosis (Becht et al., 2016). Our results pointed out the possession of higher TMB in the high-risk group (Figure 4C), with higher TMB in the high-risk group, which partially accounts for the worse prognosis (Figure 4D).

### Four cuproptosis LncRNA clusters identified by unsupervised learning

**FIGURE 5:**
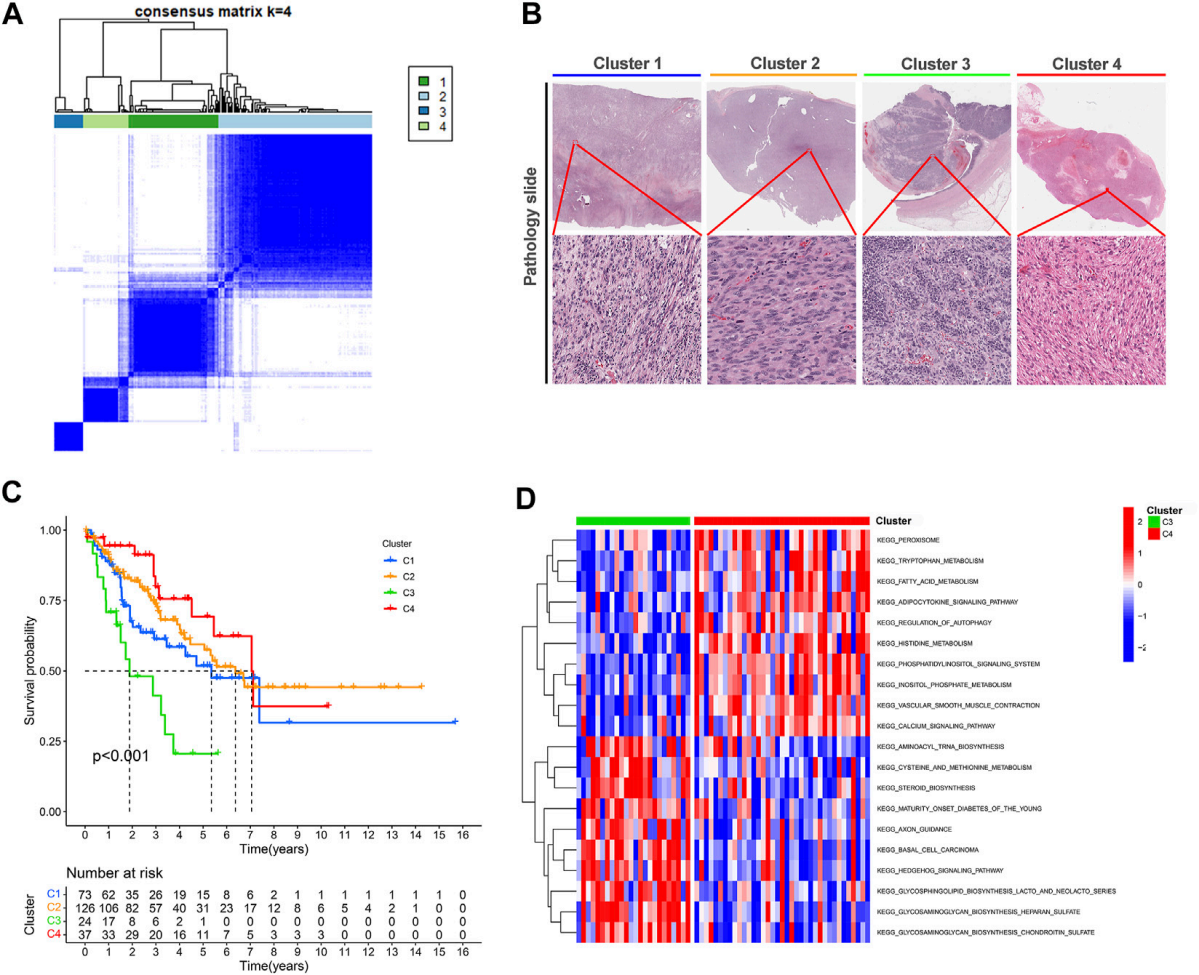
Unsupervised learning for cuproptosis lncRNA classification. **(A)** Unsupervised clustering of six cuproptosis lncRNAs and the optimized consensus matrix with *k* = 4. **(B)** Representative images of the pathological HE staining of the four clusters in TCGA-SARC. **(C)** Kaplan-Meier Curves for differential survival of four cuproptosis lncRNA clusters in the TCGA-SARC. **(D)** The KEGG enrichment levels comparison between cluster 3 and cluster 4.

**FIGURE 6:**
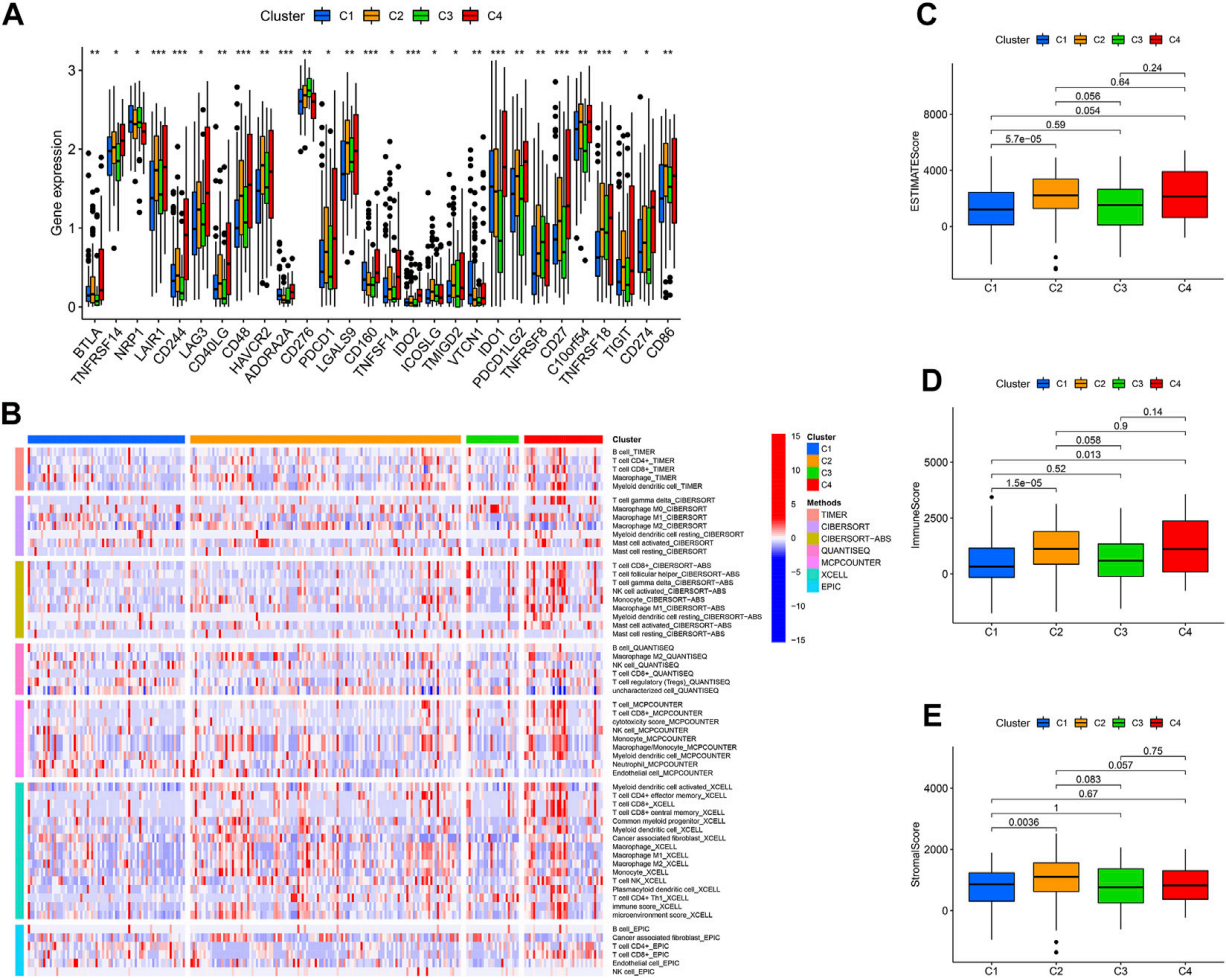
The immune landscape of four cuproptosis lncRNA clusters. **(A)** Variable expression of immune checkpoint-related genes in different clusters. **(B)** The enrichment levels of immune cells in different clusters (All *p* values <.05). The estimate score **(C)**, immune score **(D)**, and stromal score **(E)** were in the four clusters. *p < .05, **p < .01, ***p < .001.

Categorizing patients to draw a line between their unique immune statuses would allow for easy clinical guidance in tailoring individualized immunotherapy regimens. Hence, we classified the TCGA-SARC into four new clusters using an unsupervised algorithm, nominated cluster 1, cluster 2, cluster 3, and cluster 4 (Figures 5A, B). Kaplan-Meier analysis showed prognostic differences between them. Patients in cluster 3 had the worst prognosis, while those in cluster 4 had the best (Figure 5C). To further investigate the intrinsic biological differences between the different phenotypes to clarify differences in prognosis, we undertook a GSEA analysis. Doesn’t surprise us that were differences in pathway enrichment alterations between phenotypes, such as cluster 3 mainly enriched in aminoacyl tRNA biosynthesis, while cluster 4 mainly enriched in peroxisome (Figure 5D). Furthermore, the enrichment analysis of GO and KEGG displayed the intrinsic biological traits of the 4 clusters (Supplementary Figure S2). The analysis of TME cell infiltration revealed significant variations in the level of immune cell infiltration between the four clusters and cluster 4 seems to benefit from a wider infiltration of immune cells (Figure 6B). In a context where immunotherapy is searingly popular, the expression of immune checkpoint genes contributes to steering the option of immunotherapy modalities. We found variable expression of immune checkpoint genes in different clusters, where CD276 (also named PD-L1) was significantly differentially expressed in C3 and C4 (Figure 6A). Furthermore, the estimate score (Figure 6C), immune score (Figure 6D), and stromal score (Figure 6E) all display remarkable differences between C1 and C2.

### Interlinkage of CuLncScore and cuproptosis lncRNA clusters

**FIGURE 7:**
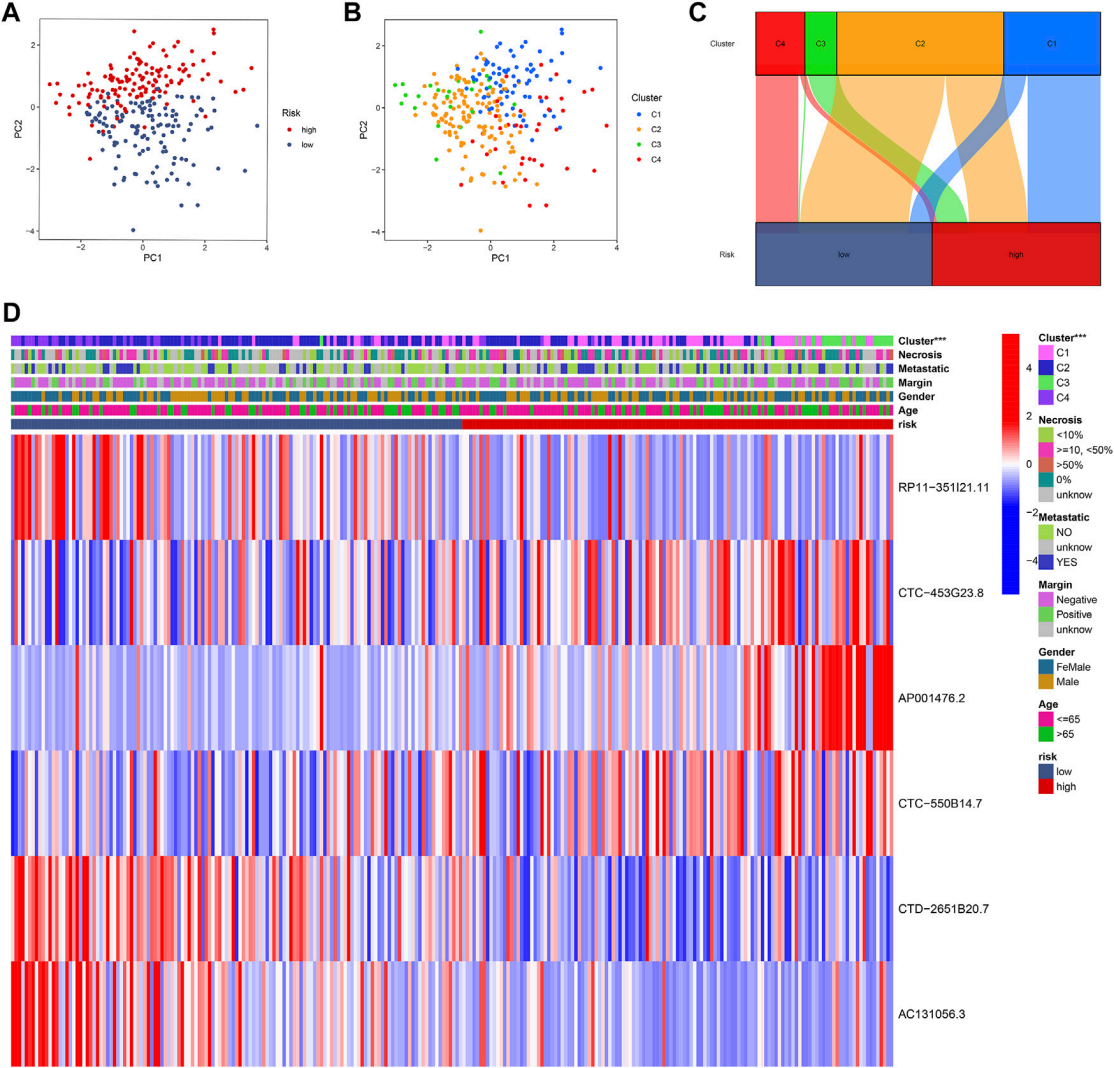
Intrinsic crosstalk between CuLncScore model and cuproptosis lncRNA clusters. Principal component analysis (PCA) plots of patients with expression profiles in different risk groups **(A)** and clusters **(B)**. **(C)** Cross-connection of patients in distinct risk groups and clusters. **(D)** Heat map of clinical characteristics of two risk groups and four clusters. ***p < .001.

The principal component analysis (PCA) plots present the distribution of patients in different CuLncScore groups (Figure 7A) and four cuproptosis lncRNA clusters (Figure 7B). The intrinsic cross-linkage between them can be traced from Figure 7C, with C3 mainly distributed in the high-risk group and C4 mainly concentrated in the low-risk group. Moreover, by comparing the clinicopathological characteristics and expression patterns of the 6 lncRNAs in separate CuLncScore and multiple Cuproptosis lncRNA clusters, we realized that the sub-clusters differed significantly between high and low-risk subgroups (Figure 7D).

### Clinical validation of CuLncScore and cuproptosis lncRNA clusters in Taizhou and TARGET cohort

**FIGURE 8:**
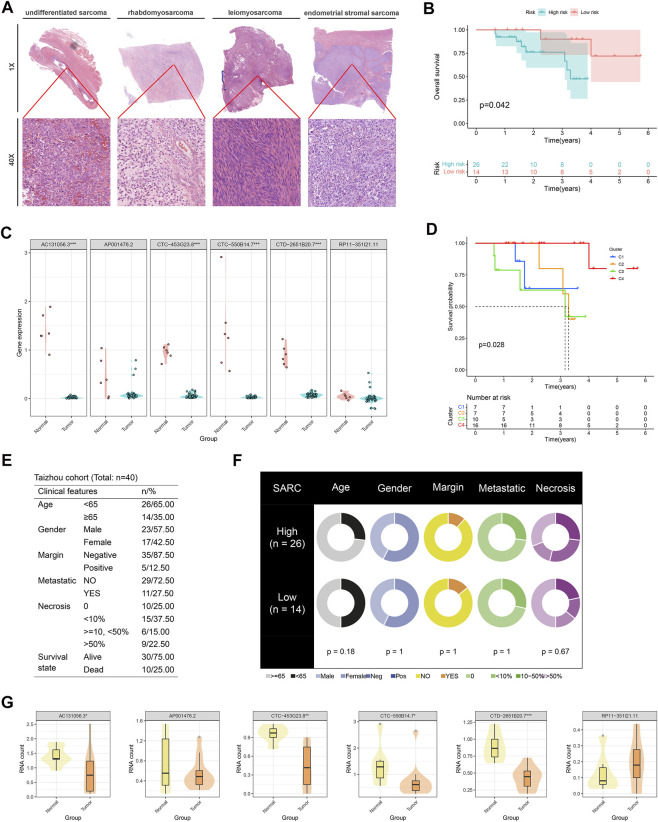
Independent queue validation of the CuLncScore model and cuproptosis lncRNA classification. **(A)** Representative images of the pathological HE staining of the Taizhou cohort. **(B)** Overall survival differences between high and low-risk groups in the Taizhou cohort. **(C)** Some sensible cuproptosis lncRNAs were identified by the RT-PCR in the Taizhou cohort. **(D)** Overall survival differences between four cuproptosis lncRNA clusters in the Taizhou cohort. **(E)** Table of clinicopathological characteristics of the Taizhou cohort. **(F)** Clinicopathological features of the distinct risk groups. **(G)** The expression distribution of cuproptosis lncRNAs in normal and tumor tissues was checked by transcriptome sequencing. *p < .05, **p < .01, ***p < .001.

*Via* profiling of the data, we constructed a neoteric CuLncScore system with superior prognostic predictive capabilities. This called for more practical testing, so we enrolled a cohort containing 12 cases of sarcoma (Figure 8A, HE pathology slide) and 6 normal mesenchymal tissue, denoted as the Taizhou cohort, for second-generation transcriptome sequencing. In our cohort, AC131056.3, CTC-453G23.8, CTC-550B14.7, and CTD-2651B20.7 were statistically markedly downregulated in the tumor, whilst AP001476.2 and RP11-351I21.11 were not notably altered (Figure 8C). This result was vouched for by qRT-PCR (Figure 8G). Patients were divided into high and low-risk groups based on the median score of CuLncScore model, and we have witnessed that those in the high-risk group had an unfavorable prognosis (Figure 8B). This outcome reaffirms the stability of CuLncScore. Other than that, Figure 8F highlights the clinicopathological characteristics of the various risk sets. Taking it further, based on the expression distribution of the 6 Cuproptosis lncRNAs, we grouped patients into 4 clusters to detect the distinct prognosis as well and showed that patients in C3 possessed the worst prognosis (Figure 8D), which was in line with the findings of TCGA-SARC. Eventually, Figure 8E exhibits the clinicopathological features of our Taizhou cohort. Externally, we pooled the osteosarcoma cohort from the TARGET database (denoted as TARGET-Osteosarcoma) for further validation of the CuLncScore and Cuproptosis LncRNA Clusters. Outcomes were realistic those patients classified in the high-risk group or C3 Cluster suffered a poorer prognosis (Supplementary Figure S1).

## Discussion

The newborn copper-associated form of cell death, cuproptosis, is setting off a burning wave of research that could shed new light on oncotherapy (Cobine et al., 2021). lncRNAs play an integral part in the progression and metastasis of malignancies in humans (Schmitt and Chang, 2016; Dong et al., 2021). As yet, few reports have investigated cuproptosis lncRNAs that could be both latent therapeutic targets and prognostic biomarkers in neoplasms. We focus on the critical role of cuproptosis lncRNAs in human cancers, especially sarcoma. In this work, with the identification of cuproptosis lncRNAs in sarcomas, we constructed a neo-risk scoring system to readily assess the prognosis of patients, labeled as CuLncScore, which is not only able to delineate the difference in immune microenvironment status of patients but also to discern patients’ response to partial immunotherapy and susceptibility to chemotherapeutic agents. In addition, we polled the SARC cohort in the TCGA database and confirmed four clusters of cuproptosis lncRNAs in sarcomas. Most emphatically, all of the above findings were practiced in our Taizhou cohort. Hence, we have justifiable faith that this CuLncScore and cuproptosis lncRNAs classification are of clinical real-world application.

At the outset of this work, cuproptosis lncRNA and sarcoma were the centerpiece agents. Through a literature search, no precise definition of cuproptosis lncRNA has been yet reported. Mindful of the correlation between the expression levels of mRNAs and lncRNAs, we characterized lncRNAs tightly associated with cuproptosis genes using the pearson correlation analysis method, which has been broadly applied in the field of computational genomics of tumors (Mehrpour Layeghi et al., 2021; Liu et al., 2022; Mohsenikia et al., 2022). In the sarcoma data, |correlation coefficient| > .4 and *p*-value <.001 were our selection thresholds, and we ultimately yielded 580 lncRNAs, defined as cuproptosis lncRNAs.

In this case, we noted the anomalous phenomenon that RP11-351I21.11 was highly expressed in the tumor, whereas the cox analysis showed that it might work as a protective factor. Likewise, CTC-453G23.8 and CTC-550B14.7 were hyper expressed in tumors, while a cox analysis realistically concluded that it could be a risk element. It seems to contradict the conventional notion that genes with high expression in tumors are oncogenes, instead, low expression is oncogenes. Actually, we missed an essential point that neoplasms are the outcomes of staged development. For instance, TGF-beta, which plays a part at different stages of cancer, is probably oncogenic in the very early stage and pro-cancer in the advanced (Tauriello et al., 2022). Moreover, since RNA-seq is a bulk-seq in which all kinds of cells are mixed, genes that are highly expressed as measured by RNA-seq would be molecules that are highly expressed on immune cells. One well-documented example is that patients with high CXCL11 expression tend to possess a high abundance of intra-tumor CD8^+^ T cells and CD56^+^ NK cell infiltration in the tumor, and those cells are correlated with the anti-tumor immune response, resulting in a favorable prognosis (Tokunaga et al., 2018).

Sarcoma, as one of the tumors with extremely poor outcomes (Burns et al., 2020), urgently desires novel scoring models to predict patient prognosis and provide guidance for individualized management (Vibert and Watson, 2022). More recently, Yang et al. (2022) and Han et al. (2022) developed scoring models based on cuproptosis-associated lncRNAs to measure the prognosis of osteosarcoma, which carries significant clinical implications. We doubt whether more cuproptosis-associated lncRNAs capable of predicting sarcoma prognosis. To tackle this issue, we developed a risk-scoring system consisting of six cuproptosis lncRNAs, denoted as CuLncScore. Our results revealed that those in the high-risk group had a worsened prognosis in CuLncScore. Meanwhile, in cuproptosis lncRNAs classification, the C3 group displayed the worst prognosis. This is not only attested in TCGA-SARC but also practically applied to our realistic Taizhou queue. Unfortunately, only very few records are available on the functions of these target lncRNAs. Where Fan, Y et al. revealed that dysregulated lncRNAs such as AC131056.3-001 might foster PD pathogenesis by enhancing the apoptosis of dopaminergic neurons (Fan et al., 2019). It remains to be explored whether they have a tumorigenic role.

At a time when tumor immunotherapy is in full swing, there is a consensus that differentiating the state of different immune microenvironments could better guide the choice-making of treatment modalities (Samstein et al., 2019). Sarcomas are believed to be immunostaining tumors with a low mutational load. The majority of soft tissue sarcomas are predominantly M2 macrophage infiltrated and possess an immunosuppressive phenotype, which puts only a very few patients at an advantage for immunotherapy (Morales et al., 2020; Roulleaux Dugage et al., 2021). Petitprez et al. (2020) noted that B cells were associated with sarcoma survival and immunotherapeutic response and they concluded that patients with the presence of tertiary lymphoid tissue with high B-cell infiltration enjoyed longer survival and more responsive immunotherapy There was less B-cell infiltration and increased macrophage M2 enrichment in the high-risk group in our analysis, hinting that they might have a weaker immunotherapeutic response. Similarly, the CD274 (Also available as PD-L1) expression was lowest in the C3 group, suggesting the necessity to cautiously consider the PD-L1 inhibitors.

Sarcomas typically have reoccurring driver genomic events, covering amplification, mutations, or translocations (Baumhoer et al., 2019; Jagodzinska-Mucha et al., 2021; van der Graaf et al., 2022). For instance, undifferentiated sarcomas frequently undergo recurrent copy number mutations, and deletions of oncogenes, such as TP53, RB1, CDKN2A, and CDKN2B, are common (Hames-Fathi et al., 2022). In other cases, patients with liposarcoma commonly have genetic alterations tied to the amplification of specific regions on chromosome 12q13-15, which encompasses CDK4 and MDM2 (Assi et al., 2020; Nishio et al., 2021). As the technology of sequencing proceeded, a massive amount of evidence pinpoints that different mutational traits are tightly linked to both prognosis and treatment decisions (Ren and Gu, 2017; Brady et al., 2022). In our scoring model, the inconsistent mutational profiles, somatic co-mutation profiles, and CNV alterations in separate risk groups further clarified the natural differences between the two from the genomic viewpoint.

We must admit that the present study contains certain limitations. Primarily, the validation cohort of this report is a single-center retrospective study and still awaits further validation by multi-center clinical cohort support. Despite the implementation of several immunohistochemical experiments, our findings imply that certain cuproptosis lncRNAs may play an essential role in sarcoma, but more in-depth mechanisms lack exploration. Our team is handling further diligent work on this topic.

## Conclusion

Profoundly, the CuLncScore model and classification system based on cuproptosis lncRNAs are excellent in assessing the prognosis and immune microenvironment status of sarcoma patients and hold prospective for further clinical application.

## Data Availability

The data presented in the study are deposited in the GEO repository, accession number GSE218806.

## Supplementary material

SUPPLEMENTARY FIGURE S1 Validation of the CuLncScore Model and the Cuproptosis LncRNA Clusters for the TARGET Cohort. **(A)** Overall survival differences between high and low-risk groups in the TARGET-Osteosarcoma cohort. **(B)** Overall survival differences between four cuproptosis lncRNA clusters in the TARGET-Osteosarcoma cohort.

SUPPLEMENTARY FIGURE S2 Biological features of 4 Cuproptosis LncRNA Clusters. GO enrichment analysis in the C1 group, **(A)** C2 group **(B)**, C3 group **(C)**, and C4 group **(D)**. KEGG pathway enrichment analysis in the C1 group **(E)**, C2 group **(F)**, C3 group **(G)**, and C4 group **(H)**.
