# Supplementary figures and images for: Cuproptosis-associated lncRNAs discern prognosis and immune microenvironment in sarcoma victims
Source: Front Cell Dev Biol. 2022 Dec 16;10:989882. doi: 10.3389/fcell.2022.989882 (PMC9800909; doi:10.3389/fcell.2022.989882)

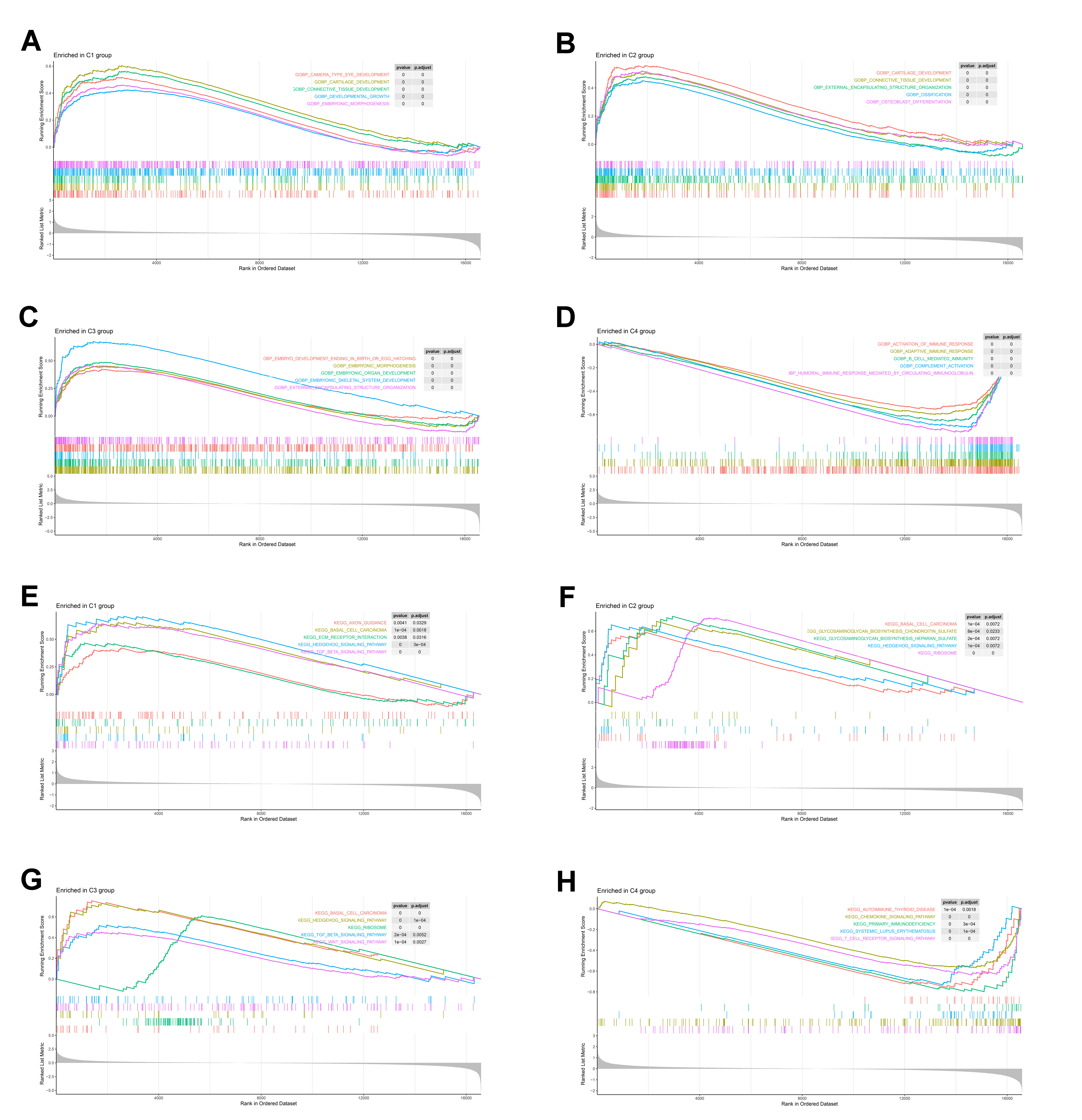

Supplement: Supplementary file 1 [file Image2.TIF]

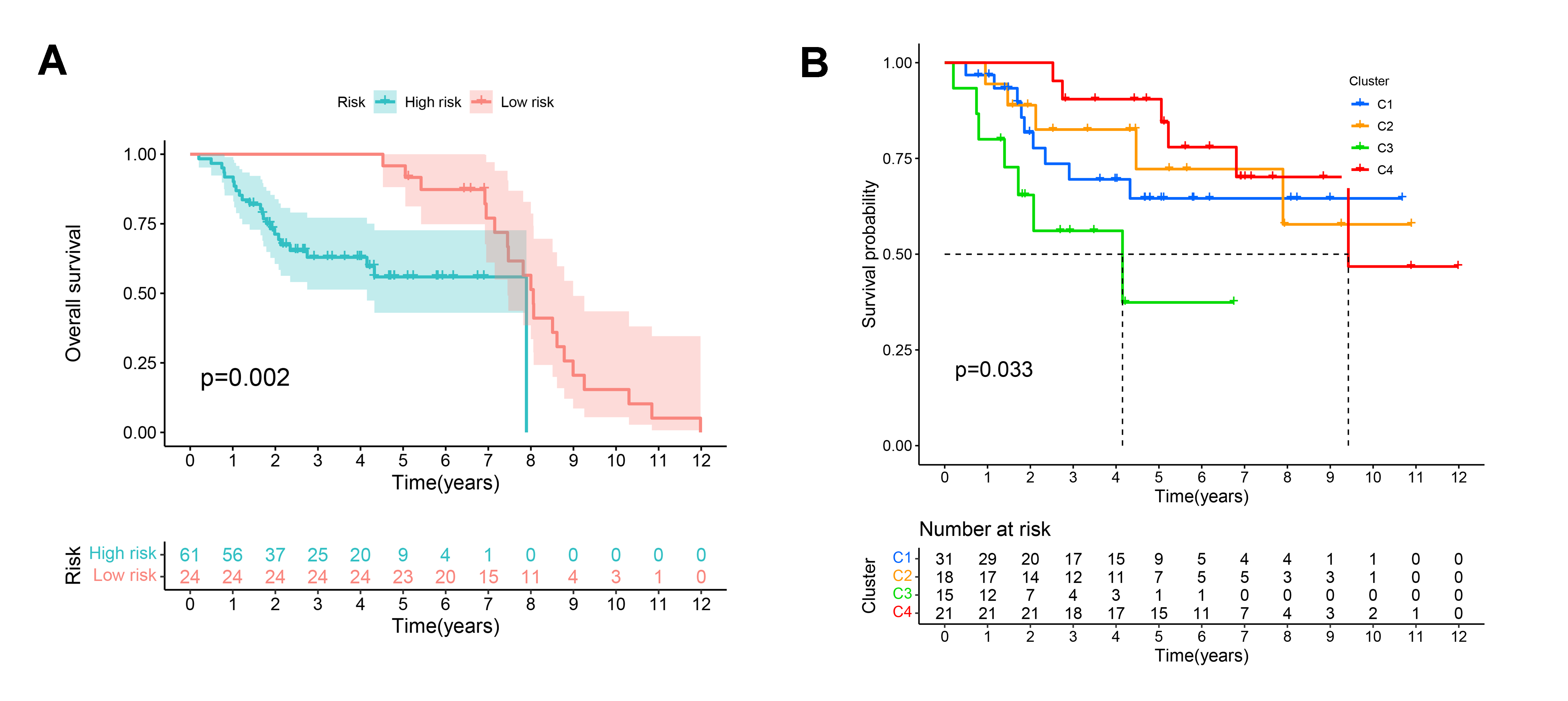

Supplement: Supplementary file 2 [file Image1.TIF]
